# Synthesis of High-Density Indium Oxide Nanowires with Low Electrical Resistivity

**DOI:** 10.3390/nano10112100

**Published:** 2020-10-23

**Authors:** Yu-Yang Chen, Shu-Meng Yang, Kuo-Chang Lu

**Affiliations:** 1 Department of Materials Science and Engineering, National Cheng Kung University, Tainan 701, Taiwan; yuyang840228@gmail.com (Y.-Y.C.); young263263@gmail.com (S.-M.Y.); 2 Center for Micro/Nano Science and Technology, National Cheng Kung University, Tainan 701, Taiwan

**Keywords:** indium oxide, nanowire, chemical vapor deposition, carbothermal, resistivity

## Abstract

In this study, indium oxide nanowires of high-density were synthesized by chemical vapor deposition (CVD) through a vapor–liquid–solid (VLS) mechanism without carrier gas. The indium oxide nanowires possess great morphology with an aspect ratio of over 400 and an average diameter of 50 nm; the length of the nanowires could be over 30 μm, confirmed by field-emission scanning electron microscopy (SEM). Characterization was conducted with X-ray diffraction (XRD), transmission electron microscopy (TEM), photoluminescence spectrum (PL). High-resolution TEM studies confirm that the grown nanowires were single crystalline c-In_2_O_3_ nanowires of body-centered cubic structures. The room temperature PL spectrum shows a strong peak around 2.22 eV, originating from the defects in the crystal structure. The electrical resistivity of a single indium oxide nanowire was measured to be 1.0 × 10^−4^ Ω⋅cm, relatively low as compared with previous works, which may result from the abundant oxygen vacancies in the nanowires, acting as unintentional doping.

## 1. Introduction

The development of nanotechnology has dramatically changed the world with electronic devices being hugely upgraded over the last decades. Different studies on various nanowires are flourishing in order to fulfill the need of the technology [1,2,3,4]. Among them, indium oxide has gained lots of attention. Indium oxide is a transparent semiconducting oxide (TSO) with a large band gap; however, the exact value of the band gap is still under debate [5]. Undoped indium oxide has an n-type semiconductor behavior due to the oxygen vacancies that naturally exist in indium oxide. These unique properties make indium oxide an appealing material to be applied in various fields, such as transparent conducting oxide (TCO), indium tin oxide (ITO), field effect transistors [6], light-emitting devices [7], and ultraviolet sensors [8].

Some works of synthesizing one-dimensional indium oxide nanowires have been reported. Because of the high melting temperature of indium oxide, most of the research teams used a chemical vapor deposition method but with different precursors; for example, indium, indium arsenide, and indium oxide were used as the precursors in previous studies [6,9]. Gold was used as the catalyst in most of the works for the vapor–liquid–solid growth (VLS) route [10]. However, certain details of the growth mechanism are still unclear. The deposition temperature could be ranging from 450 to 900 °C even for the same precursor [11,12]. The relationship between morphology and different growth conditions has not been clarified. In this work, indium oxide nanowires were synthesized through chemical vapor deposition, followed by the characterization of the grown nanowires, where SEM, XRD, TEM and PL were used. Additionally, electrical resistivity measurements of single indium oxide nanowire were performed; the purpose of the study is to investigate how the growth conditions affect the structure and properties of indium oxide nanowires and have a better understanding of this material.

## 2. Materials and Methods

**Figure 1:**
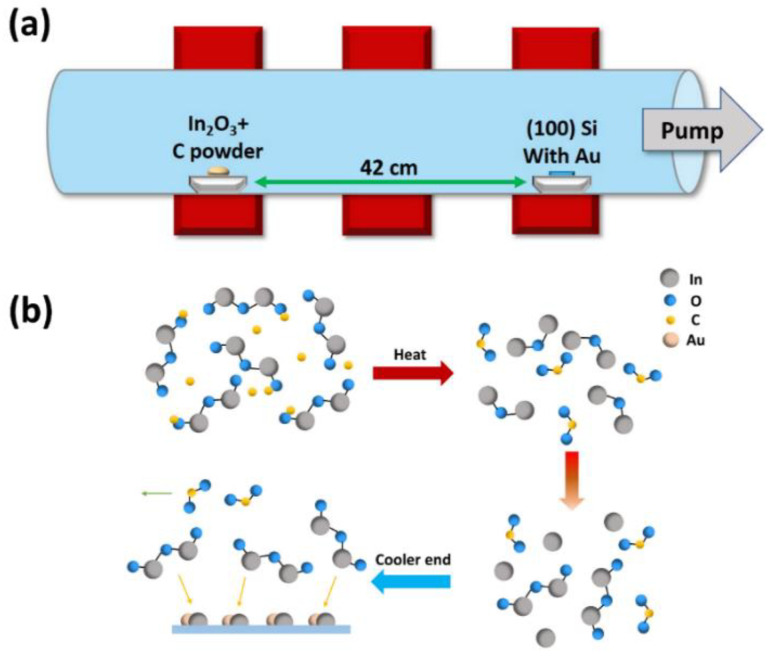
(**a**) Schematic illustration of the experimental setup, showing the placement of the precursor and substrates. (**b**) Scheme illustration of the growth mechanism; the reaction followed a vapor–liquid–solid (VLS) route.

In this experiment, indium oxide nanowires were synthesized in a conventional horizontal furnace with three individual heating zones through a chemical vapor deposition process. Firstly, (100) silicon substrates were sonicated in acetone, isopropyl alcohol and hydrogen fluoride, respectively, to remove possible contaminants, and then cleaned with deionized water. After cleaning, silicon substrates were coated with a 10 nm-thick gold film, which acted as the catalyst during the experiment. The substrates were divided into appropriate size afterward. Active charcoal (Sigma-Aldrich, St. Louis, MO, USA) was milled into powders in a mortar and mixed well with indium oxide powders (Alfa Aesar, Ward Hill, MA, USA). The mixture of active charcoal and indium oxide powders was then put into an alumina boat as the precursor and the alumina boat was placed at the center of the first heating zone of the furnace. Another alumina boat loaded with Au-coated Si substrates was placed downstream in the third heating zone of the furnace. The distance between the two alumina boats was about 42 cm, as shown in Figure 1a. To understand how different growth conditions affect the experimental results, the processing parameters were varied. The temperature of the first heating zone was set at 840~880 °C, and the temperature of the third heating zone was set at 520~580 °C. The duration of the reaction was varied from 30 to 60 min. The pressure of the system was controlled from 4 to 8 torr. No carrier gas was used during the experiment. After the reaction, the furnace was cooled down to room temperature naturally. Characterization of the grown nanowires was conducted by FE-SEM (Hitachi SU8000, Tokyo, Japan) for the morphology, grazing angle XRD (Bruker D8 discover, Fitchburg, WI, USA) for the microstructure, HRTEM (JEOL JEM-2100F CS STEM, Tokyo, Japan) for the phase identification and crystallinity and PL (HORIBA LabRAM HR, Longjumeau, France) for the optical properties. For the electrical measurements, indium oxide nanowires were placed on a previously prepared Si/SiO_2_ wafer with many independent Ag electrodes on it. Each nanowire was linked to four independent electrodes with Pt connection by a focus ion beam (FEI Nova-200 NanoLab Compatible, Hillsboro, OR, USA). A four-probe electrical measurements system was used for the I-V characteristics. Here, a special method reported by Gu et al., was utilized, being designed for measuring the resistivity of a single nanowire with accuracy and eliminating possible errors caused by contact resistance or Schottky contacts [13].

## 3. Results

**Figure 2:**
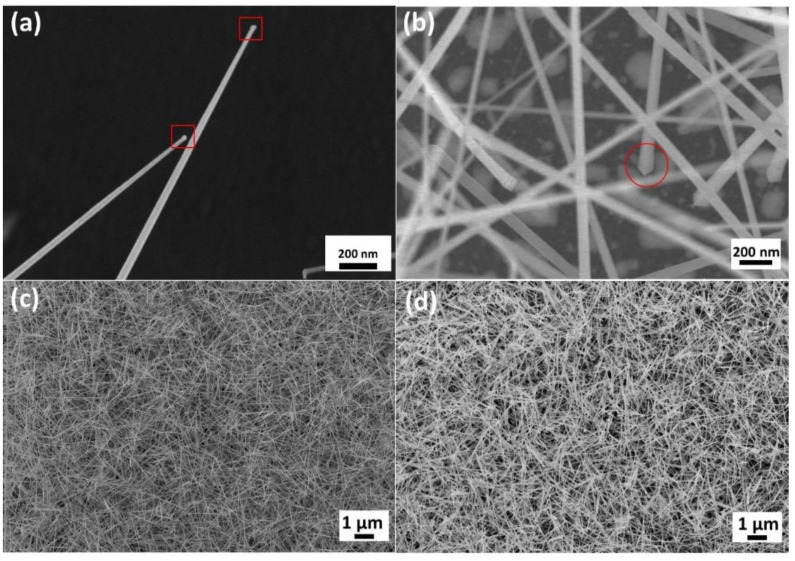
SEM images of (**a**) catalyst particles at the tip of nanowires, suggesting the VLS mechanism. (**b**) The grown nanowires have rectangular cross sections. (**c**) Indium oxide nanowires grown at the duration of 45 min. (**d**) Indium oxide nanowires grown at the duration of 60 min.

During the reaction, the precursor underwent a carbothermal reduction [1,14]. Firstly, indium oxide was reduced by active charcoal, inducing an unstable intermediate product In_2_O, and then the In_2_O was further decomposed into the more stable materials, indium and indium oxide. The indium reacted with the gold on the substrate and formed an In-Au liquid droplet. As the reaction continued, the indium in the droplet gradually reached saturation. Once it reached supersaturation, indium precipitated from the droplet and was oxidized into indium oxide. Ultimately, indium oxide nanowires were formed. Figure 1b shows the schematic illustration of the reaction. The growth mechanism followed a VLS route and could be demonstrated by the catalyst particle at the tip of the nanowires, as shown in Figure 2a. In the experiment, various parameters were tested and the morphology of the nanostructures changed accordingly. The reaction temperature affected the reaction rate of the precursor, directly influencing the concentration of the In-containing vapor. When the concentration of the vapor was too low, it was difficult for the In-Au droplet to reach supersaturation; thus, no nanowires could be observed on the substrate. Here, a reaction temperature of 860 °C was required for the growth of the nanowires. The deposition temperature also played an important role in the experiment. As the temperature was too low, the kinetic energy was not enough for forming the nanowires. In a VLS reaction, the size of catalysts had a serious impact on the morphology of the structure grown on the catalysts. With the temperature too high, the In-Au droplets tended to aggregate, forming bigger droplets; thereby, nanorods were formed instead of nanowires. It is found that 540 °C was the appropriate temperature for growing the nanowires. Since no extra gas was used during the experiment, the oxygen needed to oxidize indium came from the residual air in the tube [15,16]. As the pressure of the system was low, the amount of oxygen was not enough for the oxidation; however, at a higher pressure, the active charcoal reacted with oxygen rather than indium oxide and the carbothermal reduction failed. Finally, it can be concluded that 6 torr was the appropriate pressure for the growth of indium oxide nanowires. With the reaction time longer than 45 min, dense indium oxide nanowires were obtained, as shown in Figure 2c,d. The length of the nanowires could be over 10 μm; that of some could even be over 30 μm. The indium oxide nanowires were uniform in diameter with an average of 50 nm, having a rectangular cross section, as shown in Figure 2b. Detailed comparison of different growth conditions is provided in Figure S1 of the supporting information.

**Figure 3:**
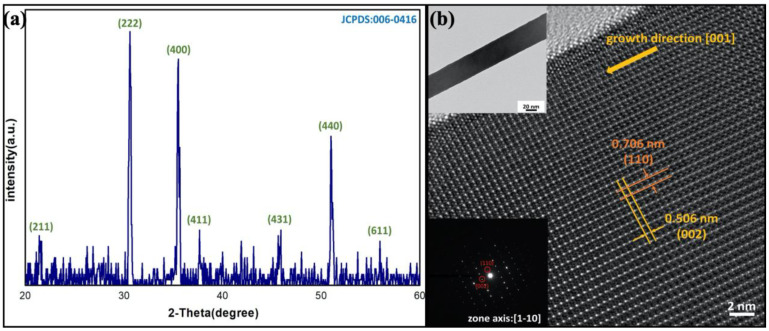
(**a**) XRD pattern of the nanowires grown at 540 °C, the peaks of which belong to indium oxide. (**b**) HRTEM image of the indium oxide nanowire, showing two interplanar spacings of 0.706 and 0.506 nm, corresponding to (110) and (002) planes. Insets are the SAED pattern and low-magnification TEM image of the nanowire.

Grazing angle XRD was performed for identifying the phase of the nanowires. Figure 3a is the XRD pattern of the nanowires, the peaks of which belong to body-centered cubic indium oxide (c-In_2_O_3_, JCPDS card no.06-0416). Additionally, no peaks of impurities were found. HRTEM was taken for further characterization of the nanowire. Figure 3b shows the HRTEM image of the nanowire. Based on the HRTEM image and selected area electron diffraction (SAED) pattern, the material has been further identified to be c-In_2_O_3_ of body-centered cubic structure (JCPDS card no.06-0416), which is coherent with the previous XRD analysis. In the HRTEM image, there are two interplanar spacings of 0.706 nm and 0.506 nm, corresponding to (110) and (002) planes. The growth direction of the nanowire is [001]. In the SAED pattern, strong diffraction from crystallographic planes, (004), (440) and (222), were observed, which are also coherent with the XRD pattern. Figure S2 is the EDS analysis for the In_2_O_3_ nanowire. The HRTEM and SEAD images indicate that the nanowires were of great crystallinity.

**Figure 4:**
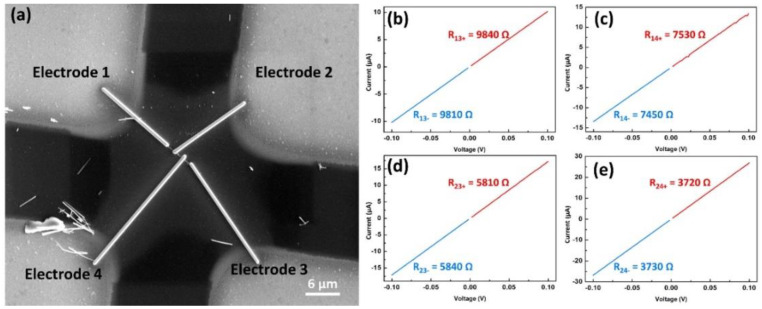
(**a**) SEM image of the nanowire under electrical measurements. The nanowire was connected to four independent electrodes by Pt connection. (**b**–**e**) Resistance measurements between different electrodes.

Figure 4 reveals the electrical resistance measurements of the indium oxide nanowire following the method introduced by Gu et al. [13]. The method requires four electrodes, as shown in Figure 4a. The resistance between different electrodes was measured; for example, R_13_ is the resistance measured by the two-contact method with one probe on the electrode 1 and the other on the electrode 3, and the results are shown in Figure 4b–e. With these data, followed by some calculations, errors from contact resistances and Schottky voltage drop could be eliminated; ultimately, the resistivity of a single nanowire was obtained. The experimental error could only result from the inaccuracy in the I–V measurement system and the leakage current through the substrate. A highly insulating substrate or a high-precision I–V measurement system may be used to improve the errors. The resistivity of the nanowire was determined to be 1.0 × 10^−4^ Ω⋅cm; the absolute highest deviation is 0.6 × 10^−4^ Ω⋅cm. Since there were few studies reporting the electrical resistivity of a single indium oxide nanowire, we also chose another material of similar composition, tin-doped indium oxide (ITO) NW, to compare with the indium oxide NW in Table 1. According to the results, it can be found that the electrical resistivity of the work is better than that of the previous studies. The lower resistivity may be attributed to a higher concentration of defects in the nanowire, which acted as unintentional doping, making the nanowire more conductive [17].

**Figure 5:**
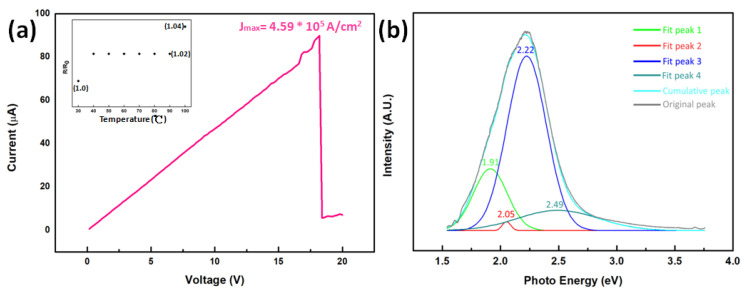
(**a**) Measurement of breakdown current density with the inset showing the temperature-dependent resistivity change. (**b**) Room temperature PL spectrum of the indium oxide nanowires, where the strongest value occurred at 2.22 eV.

Breakdown current density and temperature-dependent resistivity change were also measured, as shown in Figure 5a. The maximum current density was 5.0 × 10^5^ A/cm^2^. The resistivity from 30 to 100 °C was measured and shown in the inset of Figure 5a. The resistivity slightly rose with the increasing temperature. The resistivity at 100 °C was 4% times larger than that at 30 °C. The temperature dependence may result from the fact that the indium oxide synthesized in the experiment was a degenerate semiconductor due to the high doping concentration [23].

Figure 5b is the room temperature PL spectrum of the indium oxide nanowires, showing a wide and strong emission peak at the visible light region with the maximum peak at 2.22 eV. Based on the previous studies, peak 1 and 2 resulted from the defect luminescence of V_O_-V_In_ [23]. Peak 3 was included in the luminescence range of In_2_O_3_ nanowire [5,23,24]. Peak 4 was included in the luminescence range of the In_2_O_3_ particle [20]. All peaks in the visible light region are believed to originate from the defects in the structure of indium oxide [5,9,11,23,24,25,26]. This strong emission peak could be the evidence for the existence of defects, contributing to the lower resistivity and the degenerate semiconductor behavior. The abundant defects in the nanowires could be attributed to the lower deposition temperature in this experiment; thus, the annealing effect during cooling was weak and the defects could remain in the crystal structure. The weak peak at 1.6 eV may be the signal of the silicon substrate [27].

## 4. Conclusions

In summary, high quality indium oxide nanowires were successfully synthesized through a carbothermal reduction method. The grown nanowires were of high density and uniform diameter. The processing parameters affected the morphology of the indium oxide nanowires significantly. The optimized parameters in this experiment were the reaction temperature at 860 °C, the deposition temperature at 540 °C, the pressure at 6 torr, and the reaction time longer than 45 min. With these growth conditions, the length of the nanowires could be over 30 μm. The average diameter of the nanowire was about 50 nm and the growth direction of the nanowires was along [001]. Due to the lower deposition temperature, the as-grown indium oxide nanowires were rich in defects such as oxygen vacancies, causing the strong PL emission peak at 2.22 eV. The indium oxide nanowire possessed a very low resistivity of 1.0 × 10^−4^ Ω⋅cm. Temperature-dependent resistivity measurements indicate that the indium oxide nanowires could be a degenerate semiconductor.

## Figures and Tables

**Table 1:** Comparison of electrical resistivity with previous reports.

Structure	Resistivity (Ω⋅cm)	Reference
Thin film	9 × 10^−2^	[18]
Thin film	3 × 10^−2^	[19]
Thin film	6 × 10^−3^	[20]
Nanowire	2 × 10^−2^	[9]
Nanowire (ITO)	6 × 10^−2^	[16]
Nanowire (ITO)	8 × 10^−4^	[21]
Nanowire (ITO)	2 × 10^−4^	[22]
Nanowire	1 × 10^−4^	This work

## Supplementary Materials

The following are available online at https://www.mdpi.com/2079-4991/10/11/2100/s1, Figure S1: SEM images of indium oxide nanowires grown at different growth conditions. Figure S2: EDS analysis for the In_2_O_3_ nanowire.
