# Supplementary material for: Synthesis of High-Density Indium Oxide Nanowires with Low Electrical Resistivity
Source: Nanomaterials (Basel). 2020 Oct 23;10(11):2100. doi: 10.3390/nano10112100 (PMC7690706; doi:10.3390/nano10112100)
Supplement: Supplementary file 1 [file nanomaterials-10-02100-s001.pdf]

Supporting Information

Synthesis of High-Density Indium Oxide Nanowires with Low Electrical Resistivity

Yu-Yang Chen [1], Shu-Meng Yang [1] and Kuo-Chang Lu [1,2,*]

[1] Department of Materials Science and Engineering, National Cheng Kung University, Tainan 701, Taiwan; yuyang840228@gmail.com (Y.-Y.C.); young263263@gmail.com (S.-M.Y.)
[2] Center for Micro/Nano Science and Technology, National Cheng Kung University, Tainan 701, Taiwan
* Correspondence: gkclu@mail.ncku.edu.tw; Tel: +886-6-275-7575#62920

List of contents

Figure S1 | SEM images of indium oxide nanowires grown at different conditions.

Figure S2 | EDS analysis of the indium oxide nanowire

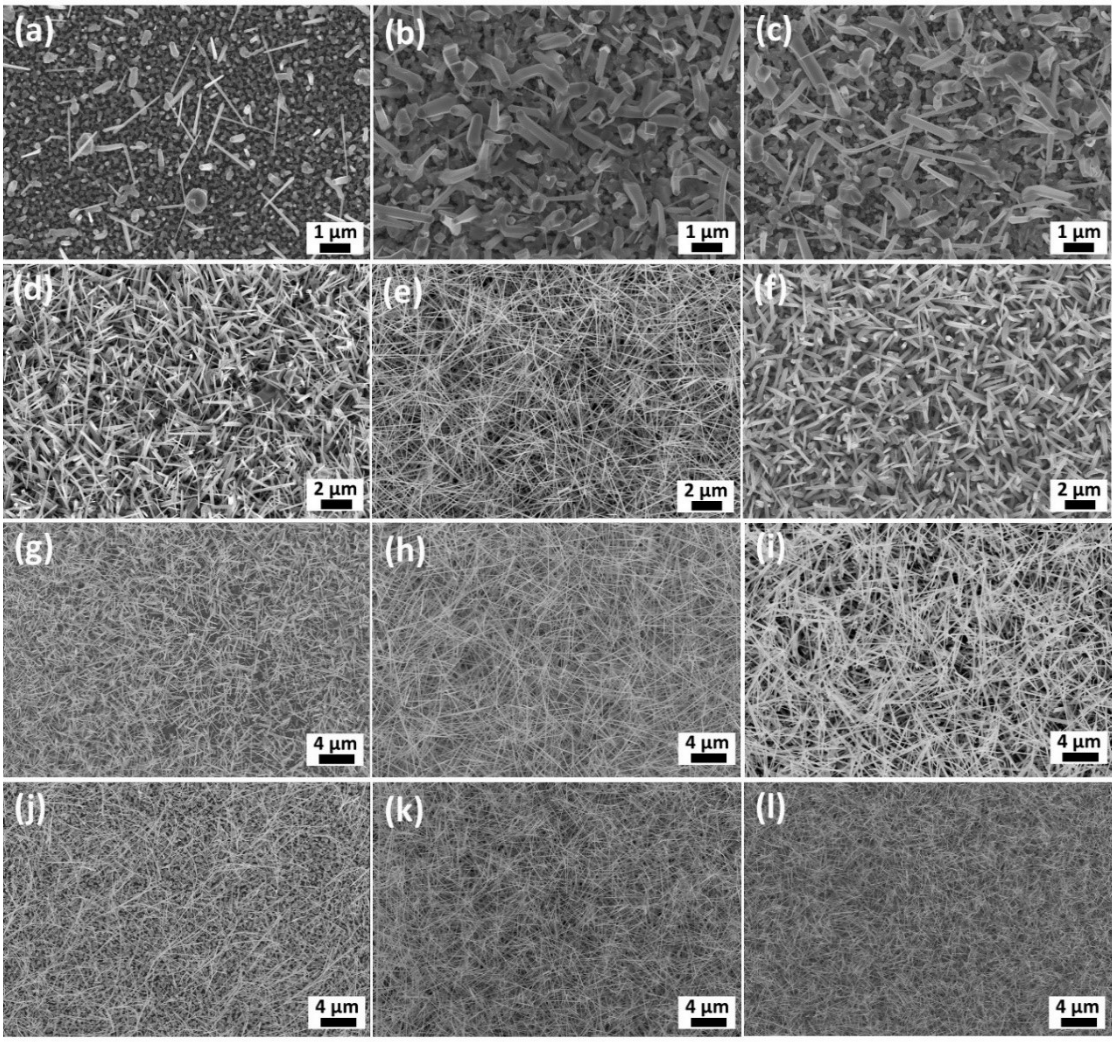

Figure S1. SEM images of indium oxide nanowires grown at different growth conditions.

Figure S1 shows SEM images of the nanowires grown at different growth conditions. Figure S1 (a-c) show the nanowires grown at different temperatures for 20 minutes. The reaction temperature for Figure S1 (a-c) was 860 ℃, 860 ℃, 880 ℃ and the deposition temperature was 540 ℃, 580 ℃, 540 ℃, respectively. Figure S1 (d-f) show the nanowires grown at 4, 6, 8 torr for 45 minutes. Figure S1 (g-i) show the nanowires grown with reaction duration of 30, 45, 60 minutes. Figure S1 (j-l) show the nanowires grown with different indium oxide weight of 0.1, 0.15, 0.2 g for 45 minutes.

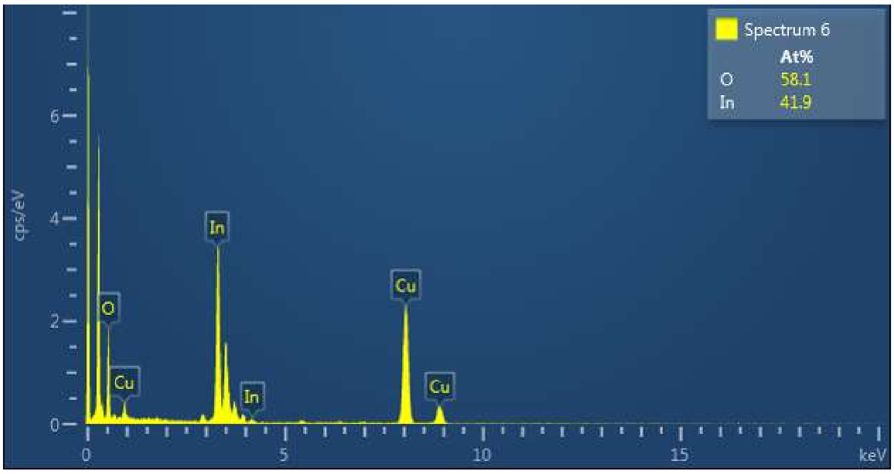

Figure S2. EDS analysis of the indium oxide nanowire.

Figure S2 is the EDS analysis for the In_2O_3 nanowire, indicating that the atomic ratio of O/In is 1.39; the deviation may be attributed to the presence of oxygen vacancies.
